# Environmental health aspects and microbial infections of the recreational water: Microbial Infections and Swimming pools

**DOI:** 10.1186/s12889-023-15183-z

**Published:** 2023-02-10

**Authors:** Faika Hassanein, Inas M. Masoud, Marwa M. Fekry, Mohamed S. Abdel-Latif, Hussein Abdel-Salam, Mohamed Salem, Amany I Shehata

**Affiliations:** 1 Department of Microbiology & Immunology, Faculty of Dentistry, Pharos University in Alexandria, Alexandria, Egypt; 2 Department of Pharmaceutical Chemistry, Faculty of Pharmacy, Pharos University in Alexandria, Alexandria, Egypt; 3 Department of Microbiology, High Institute of Public Health, Alexandria University, Alexandria, Egypt; 4 Department of Medical Laboratory Technology, Faculty of Applied Health Sciences Technology, Pharos University in Alexandria, Alexandria, Egypt; 5 Department of Water Sports Training, Faculty of Fitness Education, Alexandria University, Alexandria, Egypt; 6 Department of Tropical Health, High Institute of Public Health, Alexandria University, Alexandria, Egypt

**Keywords:** Swimming pools, Environmental health aspects, Recreational water, Physicochemical risk factors, Bather load

## Abstract

**Background:**

Swimming pools are places for practicing sports, recreation, relaxation, and socialization. However, swimming pools can expose swimmers to physicochemical and microbiological risks. Accordingly, we studied the environmental health aspects and microbial infections for such recreational water aiming to disclose the possible risks they pose on swimmers.

**Methods:**

26 pools in Alexandria, Egypt were checked for water quality; 13 pools were checked in winter then summer, and other 13 pools were checked in summer only. Water was collected from both the top and the bottom of each pool; a total of 78 samples were collected in sterile containers. Each sample was divided into three parts; the first part was used for assessing the bacteriological quality of water. They were tested for total colony count (TCC), total coliform (TC), fecal coliform, and *E. coli*. The second part was used for chemical analysis. The third part was checked for parasitological study.

**Results:**

Obtained data showed that only 7.7%, 78.2%, and 100% of the examined water samples have been found to fulfill the Egyptian standards for TCC, TC, and *E. coli*, respectively. Moreover, parasitic infection (PI) was noticed in 73.1% of the collected water samples; mainly *Cyclospra* and *Isospora* (37.2% each), followed by *Cryptosporidium* spp., *Giradia lamblia*, *Microsporidia* spp., and *Blastocystis* spp. (34.6%, 21.8%, 15.4%, and 14.1%, respectively). *Acanthameba* spp. was detected but at a lower rate (5.1%). The frequency of cleaning the swimming pools, flow rate, Cl_2_, and total dissolved solids are significantly affected PI, independently.

**Conclusion:**

The tested water samples don’t meet Egyptian bacteriological criteria. High parasitic contamination despite high residual chlorine level mainly intestinal coccidia, *G. lamblia*, microsporidia, and *Blastocystis* spp. Thus, monitoring pool’s water quality and improving the disinfection system are mandatory. Consequently, Health education regarding hygienic behaviors before and during swimming should be included in governmental programs.

## Introduction

Swimming is considered a healthy activity, and swimming pools are places for practicing sports, recreation, relaxation, and socialization [1]. However, swimming pools can also expose swimmers to several physical, chemical, and microbiological risks [2]. Poor management of swimming pools poses a high risk of microbial infections due to fecal and non-fecal sources [3]. Fecal contamination of the water is the main microbiological risk of bathing in recreational water which may be due to accidental release of remnants of fecal matter that could remain on skin of children, old and immune-compromised people especially after improper post-defecation cleansing. So, exposure to such poor microbiological water quality may pose health risks for swimmers, causing gastro-enteritis as a result of infections with bacteria, viruses, or parasites of fecal origin [4, 5]. Centers for Disease Control and Prevention (CDC) recorded 134 outbreaks of recreational water infections in the USA from 2007 to 2008 resulting in 25,000 cases [5]. In 2017, Rice and his colleagues highlighted the importance of bacteriological indicators including total coliform (TC), fecal coliform (FC) and total colony count (TCC); for determining the microbiological quality of swimming pools [6]. Moreover, fecal contamination is one of the most important factors for measuring the microbiological quality of water in swimming pools [7]. Detecting enteric pathogens, such as *Salmonella typhi, Salmonella paratyphi, Shigella dysenteriae* and *Vibrio cholerae* is difficult and costly [8]. Therefore, they are usually replaced by detecting *Escherichia coli* (*E. coli*), a strong fecal indicator [2, 9]. Tiwari et al. (2021) reported that coupling of pathogen indicators with microbial source tracking process for targeting different fecal sources, may further strengthen the process of tracking bathing waters and deliver better understanding to protect the health of bathers [10].

Moreover, at least 208 outbreaks associated with recreational water occurred In the United States during 2015–2019 [11]. Most of these outbreaks were caused by *Cryptosporidium* spp., mainly in swimming pools, and by *Legionella*, mainly during the period from June to August [9]. In 2017, CDC revealed that at least 32 outbreaks were caused by *Cryptosporidium* spp. in the United States compared to 16 outbreaks in 2014 [12]. It is typically common that when some people swim while having diarrhea definitely will put others at risk of swallowing contaminated water [13], and accidently causing chronic persistent diarrhea among immunocompromised group of people [14]. Although *Microsporidia* spp. are currently uncommon causes of recreational waterborne disease, their role as emerging pathogens is increasingly recognized. Their small size makes them difficult to be removed using conventional water filtration techniques [15]. Additionally, recreational water could be contaminated by other parasites including *G. lamblia* and free-living amebae (FLA). *G. lamblia* causes gastrointestinal infections with certain complications including malabsorption, steatorrhea, and growth retardation in children [16]. Pathogenic and opportunistic FLA can cause serious infections in humans [17], whereas *Acanthameba* spp. can produce corneal keratitis as well as chronic granulomatous amebic encephalitis [18].

Various trace chemical contaminants have been reported to occur in swimming pools [19]. Possible sources of these chemical contaminants include the water sources, bather-derived chemicals, and pool maintenance chemicals [20]. Hence, it is necessary to frequently measure the chemical quality of pool water. The American National Standard Institute has developed certain procedures to measure the water quality in public pools, including levels of total alkalinity, calcium and magnesium hardness, total dissolved solids (TDS), pH, and free chlorine contents [21]. The noncompliance of alkalinity and calcium hardness to recommended standards poses risks. Accordingly highlight the significance of these parameters in maintaining pH, sanitizer efficacy, water balance, and clarity [21]. Furthermore, filtration and disinfection are useless without proper turnover rates; so, pathogens could not be eliminated [22]. Few types of research were conducted on pool water quality, and most of them revealed poor compliance with pool water standards, especially in Alexandria; consequently, there was a strong need to improve disinfection and cleaning procedures [23]. Therefore, the current comprehensive study was carried out to investigate the operations of most of swimming pools found in Alexandria, Egypt, by collecting water samples from the top and the bottom of the swimming pools during wet and dry seasons. Then, investigating and correlating different parameters such as physicochemical, bacteriological, parasitological, and fecal indicators. So, aiming to scrutinize health and hygienic aspects of these pools, disclose the possible risks they pose on swimmers and try to find best ways for improvement.

## Materials and methods

### Study setting

Twenty-six pools (governmental and private) from 14 clubs in Alexandria, Egypt, were examined during the period from November 2020 to August 2021. Thirteen pools were double-checked, once in winter and the second time in summer while the remaining 13 pools were examined in summer only. The study was approved by the ethics committee of the High Institute of Public Health, Alexandria University in Alexandria. (HIPH556).

### Physicochemical aspects

The data of physicochemical aspects of each pool examined were obtained from pool maintenance office. Data included: the type of disinfectant used, filter capacity and cycling, turnover, pH, temperature, Pool length, water volume, work duration, and bathers’ load.

### Sample collection

Two water samples were collected from each pool during each examination: one from the top (50 cm below the surface) and one from the bottom (50 cm above the floor). So, a total of 78 water samples were collected in sterile containers.

### Water sampling for chemical analysis

Samples were collected in sterile containers (100 ml each). The samples were transferred to the Drug Research Center in Pharos University in Alexandria to be processed.

### Water sampling for bacteriological analysis

Samples were collected in sterile well-labeled one-liter ground glass stoppered bottles. These bottles contained sodium thiosulfate (100 mg/l) (Na2S2O3, sodium thiosulfate pentahydrate, 106509, Merck KGaA, Darmstadt, Germany) to dechlorinate water samples. The samples were transported within an hour using insulated coolers (4°C) to the Microbiology laboratory at the High Institute of Public Health, Alexandria University. Samples were processed immediately after arrival at the laboratory.

### Water sampling for parasitological analysis

Each sample was collected in two 5L containers. The samples were transferred to the High Institute of Public Health, Alexandria University, for immediate processing.

### Water analysis

All methods were performed in accordance with the Declaration of Helsinki.

### Physicochemical analysis

pH, total alkalinity, total dissolved solids (TDS), total hardness (TH), and chloride were analyzed physicochemically according to conventional techniques to determine the water quality.

The pH was measured using a standard pH meter HANNA instrument; total alkalinity using methyl orange, TDS using standard methods [24] Martini instrument (Mi 170 Bench Meter), while total hardness (Ca and Mg) by EDTA titrimetric method [25], and chloride content by Mohr’s method [26].

### Biological tests

#### Bacteriological analysis

Each sample was vigorously shaken and mixed before bacteriological examination.

##### Total aerobic colony count (TCC)

The TCC agar media was prepared according to the manufacturer’s instructions and poured into petri dishes. It was then incubated at 37°C for 18 hrs. to assure its sterility. The samples were 10-fold serially diluted in sterilized peptone water. Then, 100 µl of each sample and/or appropriate dilutions were plated onto the TCC agar and surface spread using a sterilized glass spreader for uniform inoculation. Next, the plates were incubated at 37°C for 48 hrs. Following an appropriate period of incubation, all visible colonies were counted, and the results were calculated by multiplying the number of colonies on each plate by the reciprocal of the dilution factor, and then multiplied by 10, which were reported as colony-forming units per milliliter [27].

#### Parasitological analysis

Water samples transferred to the Tropical Health Laboratory were filtered through sterile 0.45 µm membrane filters (Sartorius Stedim Biotech, Germany) within 2 hrs of collection. Two membrane filters were used for each sample. Three thin smears were prepared from each first membrane filter then leftovers, air-dried, and fixed. Smears were stained with Modified Ziehl Neelsen (MZN) [24], quick hot Gram chromotrope [28] and trichrome stains [29] for detecting intestinal coccidia, Microsporidia spp. and intestinal protozoa, respectively. Stained smears were examined under a light microscope using 1000× magnification. Afterward, the same filter membrane was covered by 10 ml formalin (10%) and stirred; dissolved formalin residues were collected and centrifuged [24], to be examined under a light microscope using 400× magnification

The second membrane filters were inverted on 1.5% non-nutrient agar seeded with inactivated *E. coli* in glass petri dishes for detecting *Acanthameba* spp. Monoxenically [30]. The culture plates were incubated at room temperature and monitored for trophozoites and/or cysts daily for up to two weeks by inverted phase-contrast microscope (CK; Olympus, Tokyo, Japan) using 10×and 40×objectives lens. The confirmation of *Acanthameba*, based on size and the presence of acanthopodia and a large central karyosome, was conducted using trichrome-stained smears which were examined microscopically at 1000× magnification under a light microscope [31].

#### Fecal indicators

Determination of total coliform (TC), fecal coliform (FC), and *E. coli* by the multiple tube fermentation technique as follow:

### Presumptive phase

A multiple tube dilution method was conducted for the TC and FC using lauryl sulphate tryptose broth (LST). Three rows of five Durham fermenting tubes were used. Each tube of the first raw had a 10 ml double-strength LST medium, whereas the other tubes contained a 10 ml single-strength LST medium. The samples were vigorously shaken and each fermentation tube of the first, second and third raw was inoculated with 10, 1, and 0.1 ml of the sample, respectively. The tubes were gently shaken and incubated at 35°C ± 0.5°C. After 24 ± 2hrs., the tubes were examined for the presence of gas and turbidity. Negative tubes were re-incubated for another 24 hrs. at 35°C ± 0.5°C. All positive presumptive tubes showing gas and turbidity within 48 ± 2hrs were submitted to the confirmed phase [27].

### Confirmed phase

Two sets of fermentation tubes containing brilliant green lactose bile broth were racked in the same manner as the positive presumptive tubes. The positive tubes were gently shaken, and two loopfuls from each tube were transferred into its corresponding confirmed tubes using a sterile metal loop. One set of tubes was incubated at 35°C ± 0.5°C, whereas the other was incubated at 44°C ± 0.5°C in a covered water bath. The tubes that showed gas and turbidity at any time within 48 hrs. were considered positive for TC (first set) or FC (second set). The most probable number (MPN) was calculated from the MPN tables and recorded as MPN/100 confirmed TC or FC, respectively [27].

### Completed phase

Finally, a complete test for *E. coli* was conducted. Positively confirmed tubes for FC were streaked on eosin methylene blue plates and incubated for 24 hrs. at 37°C. Typical dark-centered nucleated colonies were subjected to Gram stain, triple sugar iron (TSI), and indole, methyl red, Voges Proskauer (VP), and Simmon citrate (IMViC) tests. *E. coli* was identified as gram-negative non spore forming rods. The TSI result of *E. coli* was acid slant/acid with gas but without H2S. The IMViC results for *E. coli* were positive for indole and MR, but negative for VP and citrate tests [27].

### Statistical analysis

Data were entered, verified, and analyzed using SPSS version 25.0 (IBM, Armonk, USA). Differences and associations were tested using Pearson’s chi-squared or Fisher’s exact test, and the odds ratio (OR) with its corresponding 95% confidence interval (CI) was calculated to identify infection or association predictors. Multivariate logistic regression was conducted for establishing the relationship between independent variables (physicochemical parameters) and dependent factors (parasitic or bacterial water contaminants) to predict categorical variables.

## Results

### Physicochemical analysis

Table 1 shows the physical and chemical aspects of water samples collected from the swimming pools, presented as mean ± SD. Residual chlorine was 2.68 ± 0.107 ppm, TDS was 1304.78 ± 48.87 ppm, pH was 6.026 ± 0.0835, alkalinity was 206.56 ± 9.34 mg/l, TH was 457.69 ± 10.40 mg/l, and Ca and Mg were 302.08 ± 6.86 g/l and 155.62 ± 3.538 g/l, respectively.

**Table 1:** Chemical aspects of water samples collected from the studied pools

Chemical parameters of water samples (*n* = 78)	Median	Range	Mean ± SE	S.D.	Skewness ± SE	Kurtosis ± SE
**Cl**_**2**_**(in ppm)**	3	1–5	2.68 ± 0.107	1.17	0.435 ± 0.27	-0.694 ± 0.538
**TDS (ppm)**	1245	721–2350	1304.78 ± 48.87	431.614	0.906 ± 0.27	0.226 ± 0.538
**pH**	5.90	4.5–7.8	6.026 ± 0.0835	0.73	0.516 ± 0.27	-0.306 ± 0.53
**Alkalinity (mg/l)**	212	0–477	206.56 ± 9.34	82.52	0.585 ± 0.27	2.520 ± 0.538
**TH (mg/l)**	500	300–700	457.69 ± 10.40	91.90	0.231 ± 0.27	0.056 ± 0.538
**Ca (g/l)**	330	198–462	302.08 ± 6.86	60.65	0.231 ± 0.27	0.056 ± 0.538
**Mg (g/l)**	170	102–238	155.62 ± 3.538	31.24	0.231 ± 0.27	0.056 ± 0.538

*SE* Standard error of the mean S.D. Standard deviation

### Bacteriological analysis and chemical parameters compared to egyptian standards

**Fig. 1:**
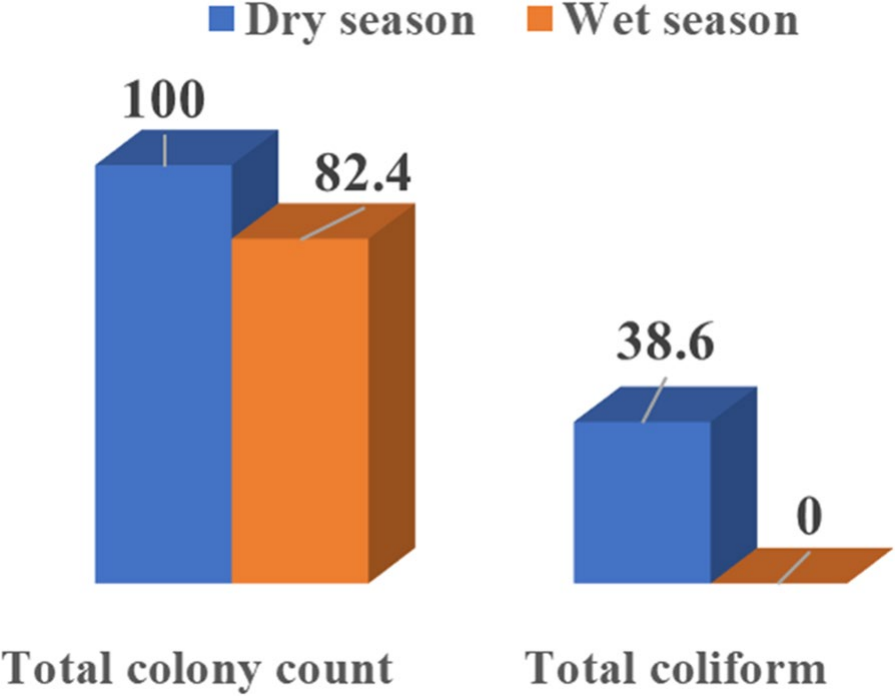
Rates of bacteriological contamination in relation to seasonal variation in collected water samples

The number of water samples that complied with the bacteriological and chemical Egyptian standards is demonstrated in Table 2. Regarding the bacteriological parameters, 7.7%, 78.2%, and 100% of the surveyed samples complied with the Egyptian standard of TCC (< 100CFU/ml), TC (0 CFU /100 ml), and *E. coli* (0 CFU /100 ml), respectively. However, only 28.2% and 11.5% of these samples were complying with the residual chlorine (1-1.5ppm) and pH levels (7.2–7.8), as determined by the Egyptian standards, respectively. Table 3 shows no difference was noted in the percentage of positive TCC (≥ 100CFU/ml) and TC (≥ 1CFU/100 ml) regarding different chlorine levels. This result shows that with residual chlorine above the Egyptian standard (> 1.5 ppm), TCC (≥ 100CFU/100 ml) and TC (≥ 1 CFU/100ml) represented 72.2% and 76.5%, respectively, compared with 27.8% and 23.5% with a standard chlorine level of 1-1.5 ppm. Table 4 shows that TCC and TC have a positive correlation between the number of swimmers and temperature; but only TCC shows a significant correlation with pH. TCC and TC were negatively correlated with residual chlorine levels; however, this was not statistically significant. Figures 1 shows that collected samples were positive for TCC in dry and wet seasons [(100% and 38.6%, respectively) and for TC in dry and wet seasons (82.4% and0.0%) respectively] with no statistically significant difference.

**Table 2:** Bacteriological and chemical parameters compared to the Egyptian standards

Bacteriological and chemical parameters	Egyptian standard	Swimming pools complying to Egyptian standards	
		N	%
**Total colony count (cfu/ml)**	**< 100**	6	7.7
**Total coliform (cfu/100 ml)**	**0**	61	78.2
***E. coli*** **(cfu/100 ml)**	**0**	78	100.0
**Residual chlorine (ppm)**	**1–1.5**	22	28.2
**pH level**	**7.2–7.8**	9	11.5

**Table 3:** Bacteriological parameters in relation to residual chlorine

Residual chlorine (ppm)	No of examined samples	Total colony count (TCC)		Total coliform (TC), n(%)	
		< 100 cfu/ml	$\ge$100 cfu/ml	< 1 cfu/100ml	$\ge$1 cfu/100ml
1–1.5	22	2(33.3%)	20(27.8%)	18(29.5)	4(23.5)
> 1.5	56	4(66.7%)	52(72.2%)	43(70.5)	13(76.5)
Total	78	6	72	61	17
Range		(30 -15$\times$10^9^)		(0–1609)	
Mean$\pm$SD		38.3 × 10^7^ ± 24.1 × 10^8^		22.72 ± 182.19	
Median		2 × 10^5^		0	
Test of significance		FEp 1		FEp 0.766	

**Table 4:** Correlation of the bacteriological indicators and certain studied parameters

Bacteriological parameters	No of swimmers		Residual chlorine in ppm		pH		Temp	
	**Rs**	***p***	**Rs**	*p*	**rs**	***p***	**rs**	***P***
**Total colony count**	0**.221**	0**.052**	**− 0.135**	0**.240**	**− 0.219**	0**.054**	0**.022**	0**.847**
**Total coliform**	0**.018**	0**.875**	**− 0.106**	0**.356**	0**.026**	0**.819**	0**.369**	0.001*

Rs Spearman’s coefficient

*P* Significant difference at *p* < 0.05

**Table 5:** Parasitic infections in relation to the physicochemical parameters of collected water samples

Parameters	Total Examined Samples	PI		*OR (95%CI)*	*p* value
		N + ve	%		
Seasonal variation					
Wet	34	22	64.7	1.000	0.147
Dry	44	35	79.5	2.121 (0.768–5.856)	
**Bather load**					
< 250	48	32	66.7	1.000	0.113
250–500	30	25	83.3	2.500 (0.806–7.757)	
**Depth of sampling**					
Superficial	39	33	84.6	3.437(1.164–10.152)	0.025^*^
Deep	39	24	61.5	1.000	
**Temperature**					
< 27 °C	20	12	60.0	1.000	0.132
≥ 27 °C	58	45	77.6	2.308 (0.778–6.842)	
**Duration of swimming pool work (in hours/ day)**					
≤ 10	46	30	65.2	1.000	0.067
> 10	32	27	84.4	2.880 (0.930–8.923)	
**Frequency of cleaning the swimming pools**					
Every 48 h.	20	19	95.0	10.0 (1.246–80.245)	0.030^*^
Daily^$^	58	38	65.5	1.000	
**Turn over (h)**					
< 6	56	39	69.6	1.000	0.281
6^$^	22	18	81.8	1.962 (0.577–6.671)	
**Flow rate (m3/min)**					
< 80^$^	24	9	37.5	1.000	< 0.001^*^
**≥** 80	54	48	88.9	13.333(4.078–43.59)	
**Cl**_**2**_**(1-1.5 ppm)**					
1-1.5^$^	22	9	40.9	1.000	< 0.001^*^
> 1.5	56	48	85.7	8.667(2.793–26.896)	
**pH (7.2–7.8)**					
< 7.2	69	51	73.9	1.417(0.320–6.26)	0.646
7.2–7.8^$^	9	6	66.7	1.000	
**Alkalinity (100–150 mg/l)**					
100–150^$^	25	17	68.0	1.000	0.489
Abnormal	53	40	75.5	1.448(0.508–4.128)	
**TDS (< 1500 ppm)**					
< 1500^$^	59	39	66.1	1.000	0.037^*^
≥ 1500	19	18	94.7	9.231(1.148–74.22)	
**Total hardness (200–400 mg/l)**					
200–400^$^	35	20	57.1	1.000	0.006^*^
> 400	43	37	86.0	4.625(1.552–13.78)	
**Total**	78	57	73.1		

***PI*** Parasitic infection, ***TCC*** Total colony count, ***TC*** Total coliform.

**FEp** Fischer - Exact significance, ***MCp***  Monte - Carlo Significance, *Significant results < 0.05, ^$^:Standard Values

### Parasitic infection (PI) and fecal indicators

**Fig. 2:**
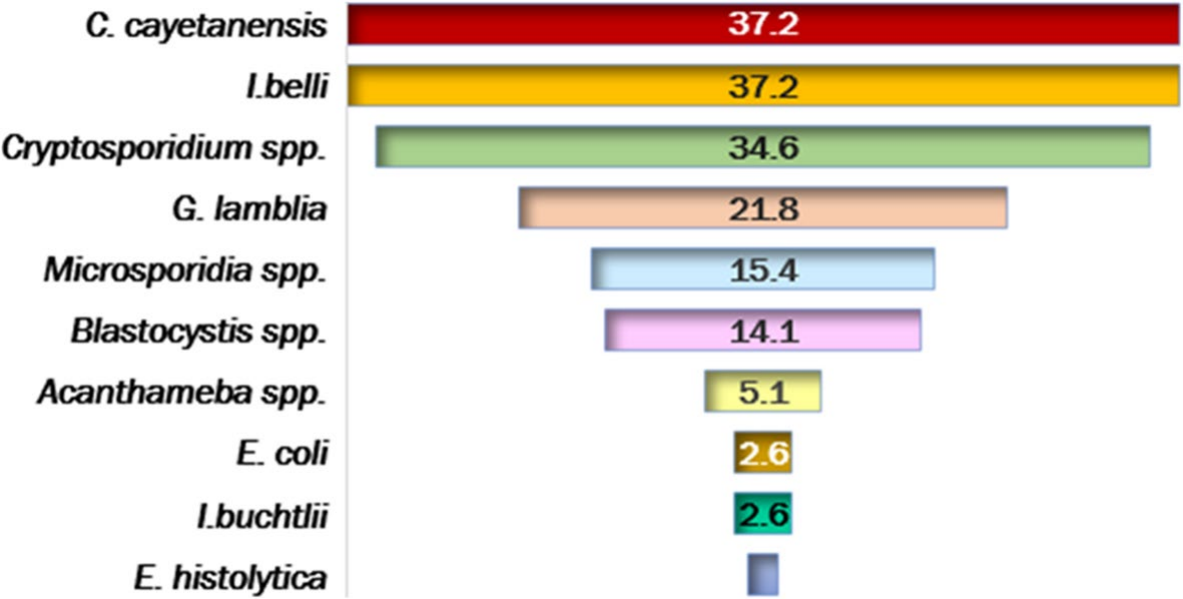
Rates of parasitic contamination in collected water samples

Table 5 shows a total PI rate of 73.1% in water samples of the studied swimming pools. It also, shows no statistically significant differences between dry and wet seasons; however, the dry season was a possible risk factor for PI, two times more than to the wet season ([79.5% vs. 64.7%] [OR = 2.121, 95% CI = 0.768–5.856]). Additionally, the odd’s ratio of PI in pools with heavy daily bather’s load (250–500 bathers /h) was twice compared to daily load of 100- <250 bathers/h ([83.3% vs. 66.7%] [OR = 2.500, 95% CI = 0.806–7.757]). Similarly, higher water temperatures (≥ 27^o^C) were two times at higher risk to harbor parasites compared to cooler water (< 27^o^ C (77.6% vs 60.0%, OR = 2.308, 95% CI = 0.778–6.842). Longer durations of swimming pool work (> 10 h/day) were almost 3 times more likely related to PI than durations of ≤ 10 hrs/day ([84.4% vs. 65.2%] [OR = 2.880, 95% CI = 0.930–8.923]). The turnover of 6 hrs was two times more likely correlated with PI that turnover of < 6 h ([81.8% vs. 69.6%] [OR = 1.962, 95% CI = 0.577–6.671]). Abnormal pH and alkalinity levels could be possible risk factors for PI compared with the normal ones ([73.9% vs. 66.7%, for pH] [OR = 1.417, 95% CI = 0.320–6.26]) and ([75.5% vs. 68.0%, for alkalinity] [OR = 1.448, 95% CI = 0.508–4.128]), respectively. On the other hand, superficially collected samples were at significant higher risk for PI compared with the deeply collected ones ([84.6% vs. 61.5%] [OR = 3.437, 95% CI = 1.164–10.152]). Regarding the frequency of cleaning the pools, it was found that the PI increased with decreasing the frequency of cleaning with a statistically significant difference (*P* = 0.030). Meanwhile, samples from highly circulated water (≥ 80 m3/min) had a high rate of PI compared to those circulated at < 80 m3/min (88.9% vs. 37.5%), and the difference was statistically significant (*P* < 0.001). Cl_2_ > 1.5 ppm was approximately nine times of higher risk for PI than Cl_2_ of 1 -1.5 ppm [(85.7% vs. 40.9) (OR = 8.667, 95% CI = 2.793–26.896)]. Similarly, TDS ≥ 1500 ppm and TH > 400 mg/l were risk factors for PI compared with TDS < 1500 ppm and TH < 400 mg/l, respectively, with a statistically significant difference. The multivariate logistic regression study for physicochemical parameters to predict the values of categorical variables affecting pools’ water contamination by parasites, showed that the frequency of cleaning the swimming pools, flow rate, Cl_2_, and TDS were significantly affecting PI independently (Table 6). As regards types of parasites, intestinal coccidia were the most prevalent parasites in samples examined (*Cyclospra* & *Isospora* 37.2%, and *Cryptosporidium* spp., 34.6%). *Acanthameba* spp. rate amounted to 5.1% while *E. coli* and *E*. *histolytica* showed the lowest rates (2.6% and 1.3% respectively) (Fig. 2).

**Table 6:** Multivariate logistic regression analysis for physicochemical parameters affecting PI

Physicochemical parameters	PI	
	P	OR (95%CI)
**Depth of sampling**	**0.077**	5.566 (0.832–37.230)
**Frequency of cleaning the swimming pools**	0.047^*^	33.565 (1.049–1074.426)
**Flow rate (m**^**3**^**/min)**	0.001^*^	30.812 (4.104–231.342)
**Cl**_**2**_	0.015^*^	10.807 (1.586–73.618)
**TDS**	0.024^*^	59.508 (1.714–2065.625)
**Total Hardness**	**0.145**	3.530 (0.646–19.284)

All variables with *p* < 0.05 following univariate analysis was included in the multivariate analysis.

*OR* Odd`s ratio, *CI* Confidence interval, *LL* Lower limit, *UL* Upper Limit, *: Statistically significant at *p* < 0.05

## Discussion

Physicochemical parameters including Cl_2_, TDS, pH, alkalinity, TH, Ca, and Mg showed great fluctuations in all the investigated swimming pools. Most samples violated the 1995 Egyptian Ministry of Health Decree no 418 standards. This was in accordance with a previous study done [23] in Alexandria in 2012 concluded that none of the surveyed swimming pools was met the Egyptian Standards for Swimming Pool Water no. 418/1995. Iranian research [32] recorded higher rates of pH, decreased Ca, temperature, and alkalinity all of them met Iranian standard. However free residual chlorine was lower and the mean TH was higher than the standard.

Most of the swimming pool water samples (92.3%) did not meet Egyptian bacteriological criteria. This conclusion is comparable with those of other studies in Alexandria, where 92.2–94.4 percent of water samples were bacteriologically unacceptable [3]. In Ghana, Amman, and Palestine, all water tests didn’t meet bacteriological requirements [33–35]. Noncompliance was due to poor maintenance, operator training, disinfection, and evaluation. In other studies, lower percentages were reported in Alexandria (43.3%), Italy (34.2%), and Greece (32.9%) [23, 36, 37]. In a study conducted a year before and during the Olympic Games in Athens 2004, swimming pool noncompliance was found to be reduced from 16% in 2003 to 0% in August 2004. This study’s outcome emphasizes the necessity of regular pool inspection [38].

Regarding the bacteriological parameters, the current study showed that 7.7%, 78.2%, and 100% of the surveyed samples complied with the Egyptian standard of TCC (< 100CFU/ml), TC (0 CFU /100 ml), and *E. coli* (0 CFU /100 ml), respectively. However, only 28.2% and 11.5% of these samples were complying with the residual chlorine (1-1.5ppm) and pH levels (7.2–7.8), as determined by the Egyptian standards, respectively. The high bacteriologically unaccepted water samples in the present study were attributed to the contamination by TCC > 100 CFU/ml, since only 7.7% of the examined swimming pool water samples complied with the Egyptian standards regarding TCC (< 100 CFU/ml). Some of the swimming pools in Alexandria showed better percentages (30.6–68%) [3].

Also, in the present study, 78.2% of water samples did not exceed the Egyptian limit for TC (0 CFU/100 ml). Lower rates were recorded in studies done in Alexandria (47%-57%) and Amman (43%) [3, 34] as well as in Iran (9.0%) and Greece (32.9%) [32, 37]. In Alexandria (2012), TC was also found in all samples and MPN/100 ml > 3.0 in 43.3% of water samples [23]. Different sample sizes, swimming pools, and disinfection techniques may explain these varying percentages.

In this study, none of the water samples examined revealed *E. coli*. However, other studies detected *E. coli* in 2.6% -8% of the samples inspected [3, 36]. In Iranian pools, the proportion was 18.2% [39]. An extremely high fecal contamination percentage was detected in Amman swimming pools, where fecal coliforms were detected in 94.7% of the samples containing TC. This shooting result was explained by a lack of pre-swimming baths, foot disinfection, and poor disinfection by untrained pool employees [34].

No difference was noted in the present study regarding the percentage of positive TCC (≥ 100CFU/ml) and TC (≥ 1CFU/100 ml) associated with different chlorine levels. This result shows that with residual chlorine above the Egyptian standard (> 1.5 ppm), TCC (≥ 100CFU/100 ml) and TC (≥ 1 CFU/100ml) represented 72.2% and 76.5%, respectively, compared to 27.8% and 23.5% with a standard chlorine level of 1-1.5 ppm. The current study revealed that TCC and TC have a positive correlation between the number of swimmers and temperature; but only TCC shows a significant correlation with pH. For years, chlorine has been the most applicable disinfectant for swimming pools. However, it must reach a certain level to be effective as a disinfectant. The accepted range of residual chlorine is 1 -1.5 ppm according to the Egyptian standard adopted by the Egyptian Ministry of Health Decree no. 418/1995. This study demonstrated that only 28.2% of the studied swimming pools’ water samples complied with the Egyptian standard regarding chlorine level, whereas 71.8% had unacceptable chlorine levels. This result was similar to those previously reported in Alexandria, where 80% -88.4% of the pools had unacceptable levels of chlorine [23, 40]. However, lower percentages (56% -61.1%) of noncomplying water samples were also reported in Alexandria by Masoud in 2016 [3]. All unacceptable chlorine levels in this study were above the permissible level (> 1.5 ppm), with a mean of 2.68 ppm. This was close to that reported previously by Hamid et al. (1993) [41], who measured the chlorine level in different swimming pools in Alexandria and found that the mean residual chlorine was 2.8 mg/l. The high chlorine level recorded in this study may explain the absence of chlorine sensitive organisms, such as *E. coli* from all water samples. Different results were reported in other studies showing lower chlorine levels among unaccepted pools’ water in Egypt and Iraq [23, 42]. Additionally, other studies have demonstrated higher and lower residual chlorine levels among noncomplying pools [34, 40]. This high variability of recorded residual chlorine levels indicates the variable amount of chlorine added to the pools (operator dependent) and lack of continuous monitoring and maintenance of pools by the authority.

However, despite high residual chlorine levels, high TCC (> 100 CFU/ml) was demonstrated in 72.2% of the studied water samples. This result may be explained by the presence of chlorine resistant bacteria (CRB). CRB are frequently described as bacteria that exhibit high resistance to chlorine disinfection or bacteria which may persist or regrow within the residual chlorine. Therefore, complete control of CRB infectivity cannot be achieved through chlorine disinfection. Another study focused on CRB pathogenicity and antibiotic resistance. Researchers found that *Mycobacterium, Bacillus, Legionella, Pseudomonas* and *Sphingomonas* are among the commonest pathogenic CRB genera, which are mainly pathogenic. However, nonpathogenic CRB were overlooked by other researchers [43, 44]. In a previous study, there was an overexpression of antibiotic resistant genes in some CRB which tolerates different chlorine levels, indicating a chlorine-associated induction of antibiotic resistance in the pathogen [43].

Mucoid Pseudomonas aeruginosa strains survive and regenerate better in chlorinated pool water than nonmucoid cultures. This was explained by overexpression of the extracellular polysaccharide layer that acts as a barrier against the disinfecting effect of chlorine [45]. In addition, Jin et al. (2020) [46] showed that chlorinated water improved plasmid transfer through genetic transformation. These transfer organelles were unaffected by chlorination, resulting in antibiotic-resistant bacteria. Environmental pollutants, CODMn, NH4+–N, and metal ions accelerated this reaction. The present study showed that 100% and 38.6% of the collected samples during the dry season, were positive for TCC and TC, respectively, whereas 82.4% and none of the samples collected in the wet season were positive for TCC and TC, respectively, with no statistically significant difference.

Globally, bathing in swimming pools is a channel for infection transmission; therefore, evaluation of parasitic infections in relation to physicochemical parameters pools’ water is mandatory [47]. It had been shown that mismanaged swimming pools may result in waterborne diseases [32]. The contamination of pools with pathogenic protozoa poses a serious threat to bathers [48]. In the current study, a PI rate of 73.1% was recorded in the water of studied swimming pools. Intestinal coccidia showed the highest rate of infections (*Cyclospra* & *Isospora* 37.2%, and *Cryptosporidium* spp., 34.6%), which may be attributed to their abilities to survive for months at ambient temperatures in moist environments [49]. This was followed by *G. lamblia*, 21.8%; *Blastocystis* spp., 14.1%; and *Microsporidia* spp., 15.4%. Lower rates of infections (≤ 2.6%) were reported for *E. coli*, and *E. histolytica.* Meanwhile, *Acanthameba* spp., rate was 5.1%.

High rates of *Cryptosporidium* spp. were detected in swimming pools in Italy and the United States (40% and 55.6%, respectively) [50, 51]. In contrast, other studies done in Italy and Netherlands revealed lower rates (28.6% and 4.6%, respectively) [52, 53]. Recently, Chalmers et al. [54] detected *Cryptosporidium* oocysts in 12/59 (20%) of pool water samples and increased to 8/12 (66%) in pool samples collected in August when bather loads were highest. The study done by Abd El-Salam in 2012 reported a low rate of both *Cryptosporidium* and *G. lamblia* (10%) in the five swimming pools selected randomly from different districts in Alexandria [23]. Meanwhile, these two parasites were detected with rates of (16.7% and 15.0%, respectively) in the 60 water samples collected from 35 swimming pools in Beijing, China [55]. Lower rates of *G. lamblia* were even detected in the Netherlands and United States (5.9% and 5.6%, respectively) [51, 53]. Contrarily, two studies done in Italy in 2004 and 2006 recorded high *G. lamblia* infection rates (40% and 28.6%, respectively) [50, 52]. The high percentage of contamination might be attributed to high bathers’ load/day, young-aged swimmers, and not having a shower before swimming, all these factors contributed to shedding of pathogens in the water [50].

Fournier et al. [56] reported *Cryptosporidia* spp. and *Microsporidia* spp. from one out of the 48 samples collected from six swimming pools in Paris, France, while giardiasis wasn’t detected. Additionally, Galván et al. (2013) [57] estimated a rate of 9% of *Cyclospra* in the different water sources examined including recreational water.

Several studies in Iran detected higher rates of *Acanthameba* spp. Than the rate reported by the present study. In 2017, Mafi [58] reported 24% as a rate for *Acanthameba* spp. in pool water and amusement parks’ pool; meanwhile Solgi et al. and Sarmadian et al. [59, 60], revealed percentages of 20% and 16.7% respectively, in swimming pools. The difference in these studies was attributed to an increase in the water temperature of hot springs.

The present work showed that dry season, heavy daily bathers’ load/h (250–500 bathers/h), higher temperatures (27C), longer duration of swimming pool work (> 10 h/day), turnover of 6 hrs, abnormal pH and alkalinity are associated with higher rate of water contamination by parasites. Superficially collected samples, decreased cleaning frequency, highly circulated water samples (≥ 80 m3/min), Cl_2_ > 1.5 ppm, TDS ≥ 1500 ppm, and TH > 400 mg/l were proved to be significant risk factors for parasitic contamination of pools’ water. Multivariate logistic regression evaluating physicochemical parameters linked with parasite water contamination revealed that the highest risk variables were pool cleaning frequency, water flow rate, Cl_2_ and TDS. The increased bather load, extended pool work hours, and infrequent cleaning, altogether, increase both fecal and nonfecal water contamination resulting in high rated of PI [61]. Zangiabadi et al. (2011) [61] reported that the simultaneous use of pools by bathers is associated with microbial contamination risk and subsequent diseases. In 2008, WHO reported that as the temperature increases to more than 27°C, microbial activity would increase accordingly [62]. Reduction in the turnover rate results in increasing the flow rate, which subsequently do not remove particles leading to lower filtration efficiency [63, 64]. In China, it was reported that the rate of *Cryptosporidium* was higher in August than in May (24.2% and 7.4%, respectively) [55]. In the done in UK, 2021, oocysts were detected in 12/59 (20%) pool water samples, 66% of them were detected in August [54]. Similar data was reported by Sheilds et al., [65] and they attributed it to a high density of bathers due to the hot weather in August in Beijing. The same was found in USA and Urmia, with higher protozoan and fungal contamination recorded during summer for the same reasons, in addition to the high temperature and humidity in this season [66, 67].

Concerning chemical parameters, this study revealed a high parasitic contamination of water in spite of the achieved desired residual chlorine level, and this could be attributed to parasitic resistance to chlorination [61, 62, 68, 69]. Hence, the desirability of physicochemical parameters could be ineffective in removing the parasites even if they were in the standard level, and this agreed with Rabi et al. [34]. A high level of TDS could reduce the chlorine efficacy and could cause dulling in pools’ water clarity [70].

In conclusion, the current comprehensive study revealed that the tested water samples don’t meet the Egyptian bacteriological criteria and about 73% of samples examined were contaminated with parasites in spite that desired chlorine level was achieved. The most common pathogens recorded were intestinal *coccidia, G. lamblia, Microsporidia spp., and Blastocystis spp. Acanthameba spp*. was also detected but at a low rate. Thus, monitoring the swimming pool’s water quality and improving the disinfection system are both mandatory. Government safety programs should incorporate health education about swimming hygiene.

## Data Availability

All data generated or analyzed during this study are included in this article.

## Abbreviations

CDC: Centers for Disease Control and Prevention
CFU: Colony forming unit
CRB: Chlorine resistant bacteria
*E. coli*: *Escherichia coli*
FC: Fecal coliform
*G. lamblia*: *Giradia lamblia*
HIV: Human immunodeficiency virus
Hrs: Hours
IMViC: Indole, methyl red, Voges Proskauer (VP), and Simmon citrate
LST: lauryl sulphate tryptose
MPN: The most probable number
MZN: Modified Ziehl Neelsen
Na2S2O3: Sodium thiosulfate
OR: Odds ratio
PI: Parasitic infection
Ppm: Part per million
SPSS: Statistical Package for the Social Sciences
TC: Total coliform
TCC: Total colony count
TDS: Total dissolved solids
TH: Total hardness
TSI: Triple sugar iron
